# Supplementary material for: Time-series transcriptome analysis identified differentially expressed genes in broiler chicken infected with mixed Eimeria species
Source: Front Genet. 2022 Aug 8;13:886781. doi: 10.3389/fgene.2022.886781 (PMC9393255; doi:10.3389/fgene.2022.886781)
Supplement: Supplementary file 2 [file DataSheet1.ZIP › 7dpi_KEGG.Gsea.1625115961950/gsea_report_for_1_1625115961950.html]

Report for 1 1625115961950 [GSEA]

| GS  follow link to MSigDB | GS DETAILS | SIZE | ES | NES | NOM p-val | FDR q-val | FWER p-val | RANK AT MAX | LEADING EDGE || 1 | KEGG\_OXIDATIVE\_PHOSPHORYLATION | Details ... | 82 | 0.79 | 3.90 | 0.000 | 0.000 | 0.000 | 1395 | tags=66%, list=12%, signal=74% |
| 2 | KEGG\_PARKINSONS\_DISEASE | Details ... | 79 | 0.69 | 3.36 | 0.000 | 0.000 | 0.000 | 1395 | tags=58%, list=12%, signal=65% |
| 3 | KEGG\_VIBRIO\_CHOLERAE\_INFECTION | Details ... | 44 | 0.62 | 2.66 | 0.000 | 0.000 | 0.000 | 509 | tags=30%, list=4%, signal=31% |
| 4 | KEGG\_HUNTINGTONS\_DISEASE | Details ... | 122 | 0.51 | 2.65 | 0.000 | 0.000 | 0.000 | 2091 | tags=52%, list=17%, signal=62% |
| 5 | KEGG\_RIBOSOME | Details ... | 72 | 0.54 | 2.60 | 0.000 | 0.000 | 0.000 | 3487 | tags=68%, list=29%, signal=95% |
| 6 | KEGG\_ALZHEIMERS\_DISEASE | Details ... | 111 | 0.45 | 2.28 | 0.000 | 0.000 | 0.001 | 1395 | tags=42%, list=12%, signal=47% |
| 7 | KEGG\_GLUTATHIONE\_METABOLISM | Details ... | 34 | 0.54 | 2.22 | 0.000 | 0.001 | 0.003 | 1388 | tags=35%, list=12%, signal=40% |
| 8 | KEGG\_CARDIAC\_MUSCLE\_CONTRACTION | Details ... | 38 | 0.53 | 2.21 | 0.000 | 0.001 | 0.005 | 1263 | tags=39%, list=11%, signal=44% |
| 9 | KEGG\_EPITHELIAL\_CELL\_SIGNALING\_IN\_HELICOBACTER\_PYLORI\_INFECTION | Details ... | 59 | 0.48 | 2.20 | 0.000 | 0.001 | 0.005 | 102 | tags=20%, list=1%, signal=20% |
| 10 | KEGG\_AMINO\_SUGAR\_AND\_NUCLEOTIDE\_SUGAR\_METABOLISM | Details ... | 38 | 0.51 | 2.14 | 0.000 | 0.001 | 0.007 | 691 | tags=32%, list=6%, signal=33% |
| 11 | KEGG\_PROTEASOME | Details ... | 31 | 0.53 | 2.10 | 0.000 | 0.002 | 0.010 | 3533 | tags=71%, list=29%, signal=100% |
| 12 | KEGG\_DRUG\_METABOLISM\_CYTOCHROME\_P450 | Details ... | 26 | 0.50 | 1.91 | 0.001 | 0.011 | 0.067 | 914 | tags=42%, list=8%, signal=46% |
| 13 | KEGG\_METABOLISM\_OF\_XENOBIOTICS\_BY\_CYTOCHROME\_P450 | Details ... | 24 | 0.46 | 1.72 | 0.011 | 0.042 | 0.258 | 914 | tags=38%, list=8%, signal=41% |
| 14 | KEGG\_PENTOSE\_AND\_GLUCURONATE\_INTERCONVERSIONS | Details ... | 11 | 0.49 | 1.44 | 0.078 | 0.201 | 0.789 | 772 | tags=36%, list=6%, signal=39% |
| 15 | KEGG\_TERPENOID\_BACKBONE\_BIOSYNTHESIS | Details ... | 12 | 0.48 | 1.43 | 0.087 | 0.200 | 0.807 | 1144 | tags=33%, list=10%, signal=37% |
| 16 | KEGG\_TYROSINE\_METABOLISM | Details ... | 23 | 0.38 | 1.36 | 0.093 | 0.277 | 0.913 | 941 | tags=35%, list=8%, signal=38% |
| 17 | KEGG\_NITROGEN\_METABOLISM | Details ... | 16 | 0.40 | 1.33 | 0.113 | 0.298 | 0.939 | 687 | tags=25%, list=6%, signal=26% |
| 18 | KEGG\_ALANINE\_ASPARTATE\_AND\_GLUTAMATE\_METABOLISM | Details ... | 22 | 0.36 | 1.28 | 0.118 | 0.353 | 0.969 | 1192 | tags=23%, list=10%, signal=25% |
| 19 | KEGG\_PHENYLALANINE\_METABOLISM | Details ... | 11 | 0.43 | 1.25 | 0.178 | 0.393 | 0.983 | 673 | tags=36%, list=6%, signal=38% |
| 20 | KEGG\_ARACHIDONIC\_ACID\_METABOLISM | Details ... | 26 | 0.33 | 1.23 | 0.158 | 0.412 | 0.989 | 881 | tags=27%, list=7%, signal=29% |
| 21 | KEGG\_TASTE\_TRANSDUCTION |  | 17 | 0.36 | 1.20 | 0.206 | 0.442 | 0.993 | 226 | tags=18%, list=2%, signal=18% |
| 22 | KEGG\_LINOLEIC\_ACID\_METABOLISM |  | 10 | 0.43 | 1.19 | 0.231 | 0.443 | 0.994 | 638 | tags=30%, list=5%, signal=32% |
| 23 | KEGG\_CYSTEINE\_AND\_METHIONINE\_METABOLISM |  | 26 | 0.30 | 1.15 | 0.232 | 0.508 | 0.998 | 673 | tags=19%, list=6%, signal=20% |
| 24 | KEGG\_PORPHYRIN\_AND\_CHLOROPHYLL\_METABOLISM |  | 19 | 0.33 | 1.14 | 0.257 | 0.514 | 0.999 | 1320 | tags=26%, list=11%, signal=30% |
| 25 | KEGG\_GALACTOSE\_METABOLISM |  | 23 | 0.31 | 1.12 | 0.271 | 0.539 | 0.999 | 691 | tags=22%, list=6%, signal=23% |
| 26 | KEGG\_CITRATE\_CYCLE\_TCA\_CYCLE |  | 28 | 0.29 | 1.10 | 0.299 | 0.587 | 1.000 | 2550 | tags=43%, list=21%, signal=54% |
| 27 | KEGG\_LYSOSOME |  | 103 | 0.21 | 1.06 | 0.287 | 0.644 | 1.000 | 974 | tags=13%, list=8%, signal=14% |
| 28 | KEGG\_ALPHA\_LINOLENIC\_ACID\_METABOLISM |  | 9 | 0.39 | 1.05 | 0.369 | 0.669 | 1.000 | 299 | tags=22%, list=2%, signal=23% |
| 29 | KEGG\_GLYCOSPHINGOLIPID\_BIOSYNTHESIS\_LACTO\_AND\_NEOLACTO\_SERIES |  | 17 | 0.31 | 1.02 | 0.404 | 0.710 | 1.000 | 827 | tags=24%, list=7%, signal=25% |
| 30 | KEGG\_PROPANOATE\_METABOLISM |  | 23 | 0.27 | 1.00 | 0.435 | 0.754 | 1.000 | 2478 | tags=48%, list=21%, signal=60% |
| 31 | KEGG\_AMINOACYL\_TRNA\_BIOSYNTHESIS |  | 20 | 0.28 | 0.98 | 0.470 | 0.788 | 1.000 | 3074 | tags=45%, list=26%, signal=60% |
| 32 | KEGG\_ARGININE\_AND\_PROLINE\_METABOLISM |  | 37 | 0.23 | 0.97 | 0.506 | 0.786 | 1.000 | 687 | tags=16%, list=6%, signal=17% |
| 33 | KEGG\_MATURITY\_ONSET\_DIABETES\_OF\_THE\_YOUNG |  | 15 | 0.30 | 0.97 | 0.504 | 0.778 | 1.000 | 1084 | tags=33%, list=9%, signal=37% |
| 34 | KEGG\_CYTOSOLIC\_DNA\_SENSING\_PATHWAY |  | 25 | 0.26 | 0.96 | 0.505 | 0.777 | 1.000 | 1237 | tags=28%, list=10%, signal=31% |
| 35 | KEGG\_DRUG\_METABOLISM\_OTHER\_ENZYMES |  | 23 | 0.25 | 0.91 | 0.597 | 0.881 | 1.000 | 772 | tags=17%, list=6%, signal=19% |
| 36 | KEGG\_GLYCOSAMINOGLYCAN\_BIOSYNTHESIS\_KERATAN\_SULFATE |  | 13 | 0.29 | 0.89 | 0.610 | 0.915 | 1.000 | 1998 | tags=31%, list=17%, signal=37% |
| 37 | KEGG\_PANTOTHENATE\_AND\_COA\_BIOSYNTHESIS |  | 12 | 0.30 | 0.89 | 0.598 | 0.892 | 1.000 | 1150 | tags=33%, list=10%, signal=37% |
| 38 | KEGG\_VALINE\_LEUCINE\_AND\_ISOLEUCINE\_DEGRADATION |  | 36 | 0.22 | 0.89 | 0.683 | 0.873 | 1.000 | 1648 | tags=25%, list=14%, signal=29% |
| 39 | KEGG\_FATTY\_ACID\_METABOLISM |  | 30 | 0.22 | 0.88 | 0.692 | 0.871 | 1.000 | 1648 | tags=33%, list=14%, signal=39% |
| 40 | KEGG\_SELENOAMINO\_ACID\_METABOLISM |  | 19 | 0.24 | 0.82 | 0.742 | 0.979 | 1.000 | 941 | tags=16%, list=8%, signal=17% |
| 41 | KEGG\_PYRUVATE\_METABOLISM |  | 30 | 0.21 | 0.81 | 0.816 | 0.973 | 1.000 | 1687 | tags=23%, list=14%, signal=27% |
| 42 | KEGG\_ETHER\_LIPID\_METABOLISM |  | 20 | 0.23 | 0.80 | 0.759 | 0.966 | 1.000 | 1430 | tags=20%, list=12%, signal=23% |
| 43 | KEGG\_RNA\_POLYMERASE |  | 20 | 0.23 | 0.80 | 0.773 | 0.946 | 1.000 | 2813 | tags=50%, list=23%, signal=65% |
| 44 | KEGG\_GLYCOLYSIS\_GLUCONEOGENESIS |  | 40 | 0.19 | 0.79 | 0.857 | 0.932 | 1.000 | 1513 | tags=23%, list=13%, signal=26% |
| 45 | KEGG\_GLYCINE\_SERINE\_AND\_THREONINE\_METABOLISM |  | 19 | 0.22 | 0.77 | 0.821 | 0.949 | 1.000 | 1415 | tags=26%, list=12%, signal=30% |
| 46 | KEGG\_GLYOXYLATE\_AND\_DICARBOXYLATE\_METABOLISM |  | 14 | 0.24 | 0.75 | 0.817 | 0.958 | 1.000 | 2364 | tags=43%, list=20%, signal=53% |
| 47 | KEGG\_LIMONENE\_AND\_PINENE\_DEGRADATION |  | 7 | 0.30 | 0.73 | 0.818 | 0.958 | 1.000 | 1648 | tags=43%, list=14%, signal=50% |
| 48 | KEGG\_GLYCOSAMINOGLYCAN\_DEGRADATION |  | 17 | 0.22 | 0.73 | 0.846 | 0.940 | 1.000 | 959 | tags=18%, list=8%, signal=19% |
| 49 | KEGG\_VALINE\_LEUCINE\_AND\_ISOLEUCINE\_BIOSYNTHESIS |  | 5 | 0.31 | 0.68 | 0.871 | 0.968 | 1.000 | 1992 | tags=40%, list=17%, signal=48% |
| 50 | KEGG\_PYRIMIDINE\_METABOLISM |  | 80 | 0.13 | 0.63 | 1.000 | 0.984 | 1.000 | 2091 | tags=21%, list=17%, signal=26% |
| 51 | KEGG\_PROTEIN\_EXPORT |  | 21 | 0.16 | 0.58 | 0.989 | 0.984 | 1.000 | 960 | tags=10%, list=8%, signal=10% |
Table: Gene sets enriched in phenotype **1 (5 samples) **[plain text format]****
